# In Vitro Wound Healing Properties of Novel Acidic Treatment Regimen in Enhancing Metabolic Activity and Migration of Skin Cells

**DOI:** 10.3390/ijms23137188

**Published:** 2022-06-28

**Authors:** Pivian Sim, Yunmei Song, Gink N. Yang, Allison J. Cowin, Sanjay Garg

**Affiliations:** 1 Centre for Pharmaceutical Innovation (CPI)Clinical and Health Sciences, University of South Australia, Adelaide, SA 5000, Australia; simpy010@mymail.unisa.edu.au (P.S.); may.song@unisa.edu.au (Y.S.); 2 Regenerative Medicine, Future Industries Institute, University of South Australia, Adelaide, SA 5095, Australia; gink.yang@mymail.unisa.edu.au (G.N.Y.); allison.cowin@unisa.edu.au (A.J.C.)

**Keywords:** acidic, buffer, ionic strength, pH, proliferation, metabolic activity, migration, skin, treatment, wound healing

## Abstract

Strategies that alter the pH of wounds to improve healing outcomes are an emerging area of interest. Currently, there is limited understanding of the effect of hydrogen (H^+^) on the functionality of skin cells during proliferation and migration, highlighting the need for research to determine the effect of pH during wound healing. This study aimed to determine the effect of acidification on the metabolic activity and migration of human immortalized keratinocytes (HaCaT) and human foreskin fibroblasts (HFF). In vitro models were used with phosphoric and citric acid buffers at a pH range between 3 and 7. Our results showed that cells were more viable in buffers with low rather than high ionic strength. A time-dependent effect of the acidification treatment was also observed with cell metabolic activity varying with treatment duration and frequency. Our results showed that a 24 h treatment and subsequent resting phase significantly improved cell proliferation and migration. This in vitro study is the first to establish a correlation between the role of acidic pH, molarity and treatment regimen in cellular activity. Our data demonstrated a positive effect of acidic pH on cell metabolic activity and migration rate, suggesting a clinical potential in indications such as wound healing.

## 1. Introduction

Under normal conditions, the outer environment of the skin maintains an acidic pH in the range of 4 to 6. When the skin barrier is damaged, the natural acidic pH of the skin is disrupted by the physiological pH of the underlying tissue. This change in microenvironment from acidic to physiological pH has been shown to affect wound healing [1,2]. Understanding how the acidification of the wound microenvironment affects different processes of healing is an emerging area of interest.

Studies have shown that decreasing wound pH by acidification improves the dynamic regeneration of wounds by supporting cellular processes within the healing cascade. Ascorbic acid, commonly known as vitamin C, has been proven to enhance dermal fibroblast proliferation and migration in vitro [3]. Retinoic acid, an active metabolite of vitamin A, has been found to elevate collagen synthesis in vitro [4]. Treatment using folic acid and its derivatives show increased DNA synthesis and repair, whilst hyaluronic acid has been reported to support extracellular matrix (ECM) synthesis by stimulating angiogenesis [5,6,7,8]. Cell migration, a key factor during re-epithelization and wound closure, is enhanced when treated with usnic acid, and this correlates to faster cellular repair in vitro [9,10]. Abietic acid shows a similar effect, presenting stimulatory effects on the migration of endothelial cells [11,12]. Syringic acid has been reported to accelerate wound regeneration by increasing cell re-epithelialization and collagen synthesis in diabetic wounds [13,14]. Additionally, fumaric acid can enhance fibroblast proliferation, granulation tissue formation, collagen synthesis and angiogenesis in vivo [15,16].

An acidic microenvironment can influence microbial growth in skin wounds by imparting an inhospitable environment for bacterial growth that neutralizes ammonia toxicity produced by skin microorganisms such as *Pseudomonas aeruginosa*, commonly present in chronic wounds [17,18]. Furthermore, the stimulative effect of acidification on wound healing has been correlated to increased oxygenation at the wound site. Reduction in wound pH by 0.6 units using chemical acidification has been shown to increase the amount of oxygen released from oxyhemoglobin by an additional 50%. Higher wound oxygen saturation is known to promote fibroblast growth, collagen synthesis and epithelialization [17]. Inducing an alkaline microenvironment in wounded tissues causes a paradoxical effect, lowering wound oxygen concentration and tension, resulting in an increase in ammonia toxicity that delays the process of epithelialization and wound closure [17,18]. Whilst acidification of the wound microenvironment is known to promote wound regeneration, there is little understanding of how different types of acids, pH values and ionic strength affect the wound healing process.

The present study utilized in vitro methods to determine the effect of acidic culturing conditions on cell proliferation and migration in keratinocytes and fibroblasts. This study aimed to establish the effect of acidic pH, molarity (ionic strength) and dosing regimen on cellular functions. The tetrazolium MTT metabolic assay and scratch migration assay were used to investigate the effect of phosphoric and citric acids, with different pH values and treatment durations, on cell viability and migration.

## 2. Results

### 2.1. Low Ionic Strength Increases Metabolic Activity of Keratinocytes

**Figure 1:**
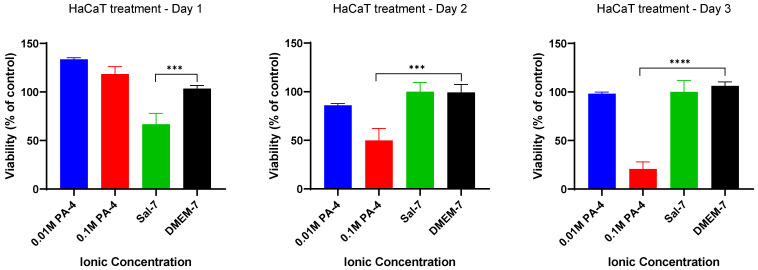
HaCaT cells metabolized well at low ionic strength and acid molarity of 0.01 M. The metabolism rate was significantly reduced with increased acid molarity to 0.1 M. Each bar represents the mean ± standard deviation. *** *p* < 0.005 and **** *p* < 0.001 indicates that the results obtained were statistically significant from those of the control group (DMEM-7).

The effect of acids’ ionic strength (molarity) on the metabolic activity of primary keratinocyte (HaCaT) cells was investigated using an MTT assay following treatment regimen A (Table 1). A 24 h acidification process over a 3 day treatment period was used to study the effect of high- (0.1 M) and low-ionic-strength (0.01 M) phosphoric acid at pH 4 (PA-4), as shown in Figure 1. The study showed that initial (Day 1) HaCaT cells remained viable in buffers with both high- and low-ionic-strength phosphoric acid, and acidification had a positive effect on cell metabolic activity (approximately 30% and 20%, respectively) in comparison to that of the control group (DMEM-7). Subsequent treatment (Day 2 to Day 3) with low-ionic-strength phosphoric acid (0.01 M) had little effect on cell viability. However, HaCaT cells treated with high-ionic-strength (0.1 M) phosphoric acid showed a significant decrease in cell viability after Day 1 treatment, from 118% to 50% (Day 2) and subsequently to 21% (Day 3). The results showed that a decrease in percentage viability of the treatment groups were not due to the treatment protocol. These findings suggest that HaCaT cells did not tolerate a high-ionic-strength acidic buffer.

### 2.2. Acidification Enhances Metabolic Activity of Keratinocytes and Fibroblasts

**Figure 2:**
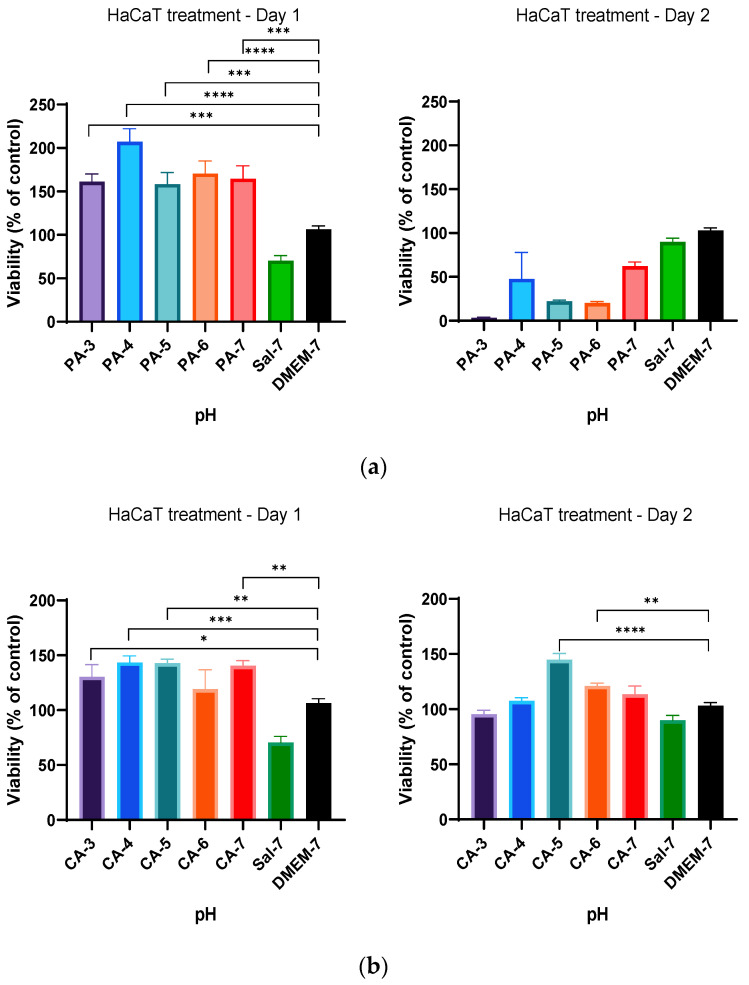
Percentage viability of HaCaT cells when treated with (**a**) 0.01 M phosphoric acid (PA) buffer solutions, with pH ranges from 3 to 7 and (**b**) 0.01 M citric acid (CA) buffer solutions, with pH adjusted to 3, 4, 5, 6 and 7. HaCaT cells tolerated well within an acidic pH range of 3 to 7 for 24 h. Each bar represents the mean ± standard deviation. * *p* < 0.05, ** *p* < 0.01, *** *p* < 0.005 and **** *p* < 0.001 indicates that the results obtained were statistically significant from those of the control group (DMEM-7).

The metabolic activity of acidified keratinocytes (HaCaT) and fibroblasts (HFF) were studied following treatment by acidification per regimen A (Table 1). This regimen consisted of continuous acidification with low-ionic-strength (0.01 M) phosphoric and citric acid buffers at a pH ranging from 3 to 7. As shown in Figure 2a, a significant increase in cellular metabolic activity for the initial 24 h treatment (Day 1) was observed in HaCaT cells when treated with phosphoric acid buffers. The rate of metabolism was measured to be significantly higher (*p* < 0.05) in all treatment groups when compared to that of the control group (DMEM-7). However, cells in media treated with further PA-3 to PA-7 acidification (Day 2) showed a decrease in metabolic activity (Figure 2a). In contrast, cells subsequently treated (Day 2) with citric acid buffers remained viable in buffers with pH 5 and 6 and showed a significant increase in metabolic activity on Day 2 (Figure 2b).

**Figure 3:**
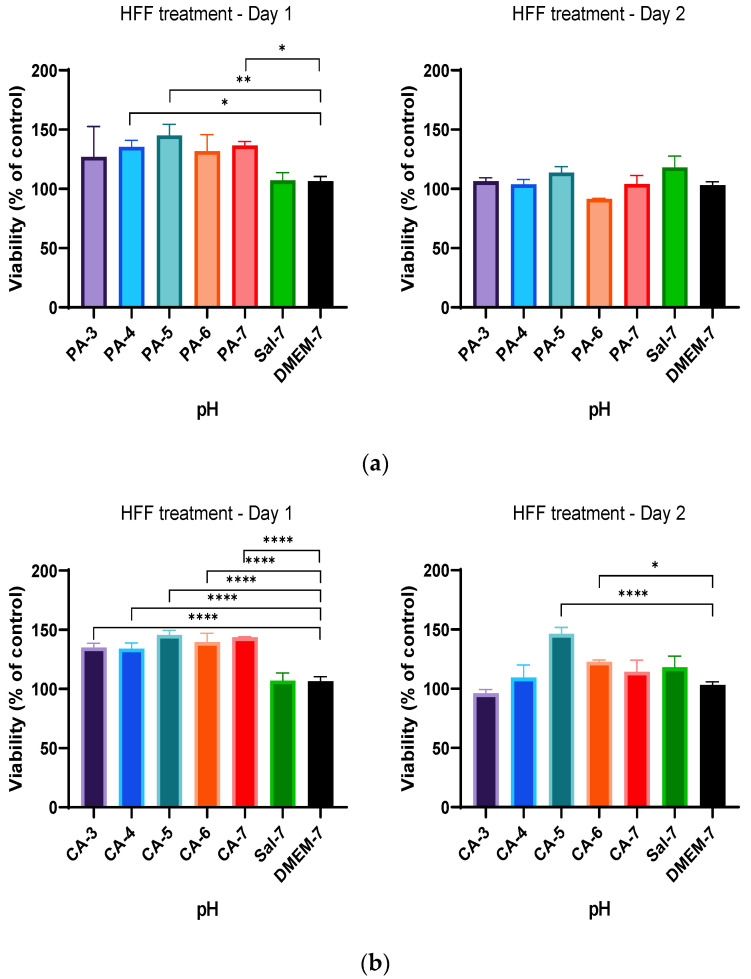
Percentage viability of HFF cells when treated with (**a**) 0.01 M phosphoric acid (PA) buffers with pH ranges from 3 to 7 and (**b**) 0.01 M citric acid (CA) buffers with pH values of 3, 4, 5, 6 and 7 utilizing the standardized MTT protocol of 24 h continuous treatment without a resting period. HFF cells metabolized well with phosphoric acid buffer treatments of pH 4, 5 and 7, while the metabolic rates of HFF cells were all statistically significant when treated with citric acid buffers at T = 24 h (Day 1). Each bar represents the mean ± standard deviation. * *p* < 0.05, ** *p* < 0.01 and **** *p* < 0.001 indicates that the results obtained were statistically significant from those of the control group (DMEM-7).

**Figure 4:**
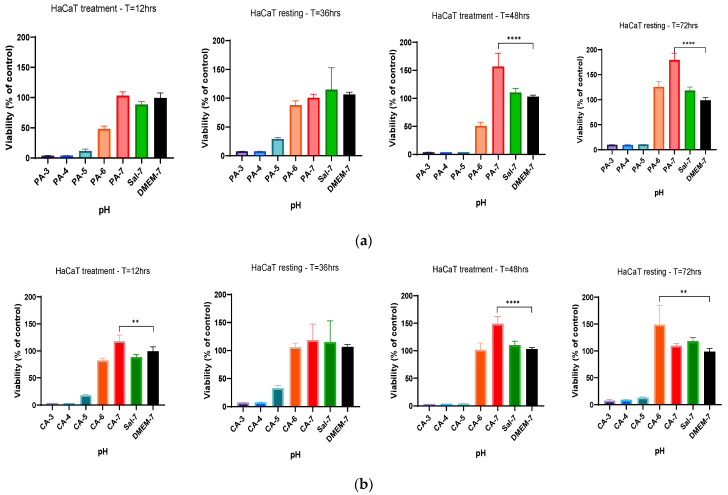
Percentage viability of HaCaT keratinocytes at different time points when treated with (**a**) 0.01 M phosphoric acid (PA) and (**b**) citric acid (CA) buffers, with pH adjusted to 3, 4, 5, 6 and 7. Each bar represents the mean ± standard deviation. ** *p* < 0.01 and **** *p* < 0.001 indicates that the results obtained were statistically significant from those of the control group (DMEM-7).

This treatment regimen was also investigated on fibroblasts (HFF) following a similar protocol. Similarly, HFF cells remained viable when treated with acidic buffers at pH ranging from 3 to 6 for the first 24 h (Day 1) (Figure 3). The results also demonstrated an increase in metabolic activity by a further 29%, 39% and 30%, respectively, when media were added with phosphoric acid with a pH adjusted to 4, 5 and 7, as shown in Figure 4a. HFF cells tolerated the change in pH and showed little changes in overall metabolic activity on subsequent citric acid treatment (Day 2) at all pH ranges from CA-3 to CA-7 (Figure 4b). Contrasting results were obtained for Day 2 treatment by both acidic buffers in both HaCaT and HFF cells.

### 2.3. Metabolic Activity Influenced by pH and Time-Dependent Acidification

To investigate if cell metabolic activity was affected by the duration of acidification, a different treatment regimen (treatment regimen B) was tested. Treatment regimen B consisted of an acidic buffer treatment for 12 h, followed by a 24 h resting interval involving replacing the acidified medium with a DMEM solution. Metabolic activity was then assessed after 72 h as described in Table 1. The results demonstrated that acidification following treatment regimen B showed that HaCaT cells did not tolerate acidification treatment with low pH 3–6 of phosphoric and citric acid buffers, demonstrating low percentage cell viability in comparison to that of the control group (DMEM-7) throughout the treatment period, as shown in Figure 4. However, better tolerance and higher viability of HaCaT cells were observed when treated with phosphoric acid (PA-6 and PA-7) and citric acid (CA-6 and CA-7) buffers at a pH range closer to physiological pH, in comparison to lower-acidic-pH-treated cells. Percentage cell viability higher by 50%was observed in HaCaT cells when treated with a phosphoric acid pH 7 buffer (PA-7) in comparison to that of control (DMEM-7), as shown in Figure 4a. An increase in metabolic activity of approximately 20% to 40% (*p*-value < 0.001) was also observed in HaCaT cells following treatment with citric acid at pH 7 (CA-7) (Figure 4b). These findings indicate that shorter treatment intervals of 12 h in the treatment protocol were not effective in increasing cell growth.

### 2.4. Treatment Regimen C Improves the Proliferation Rate

**Figure 5:**
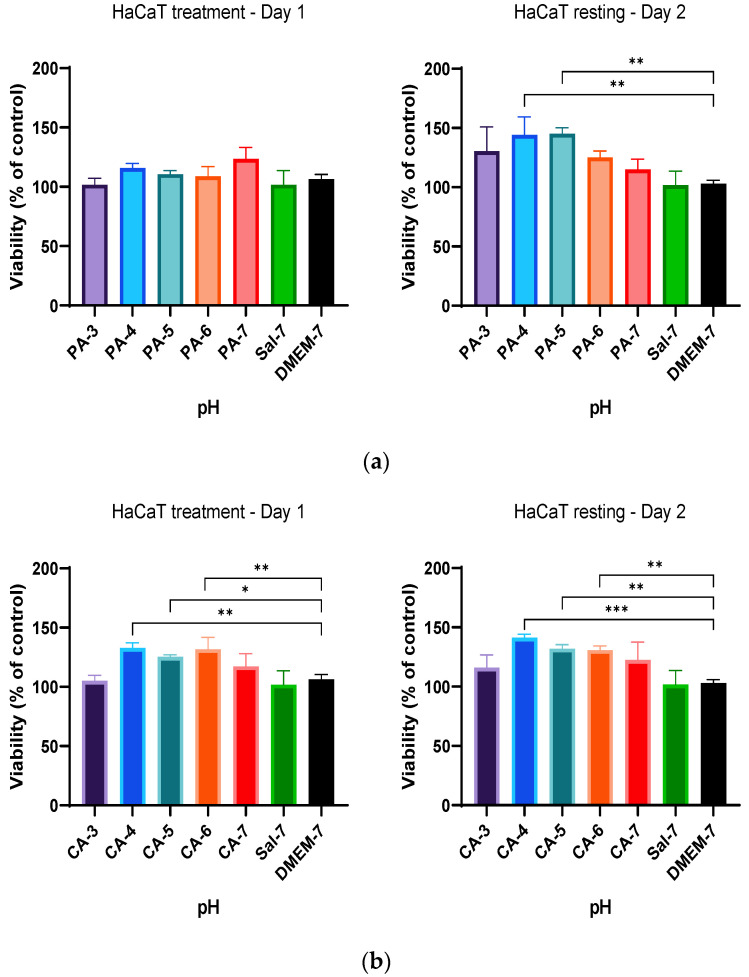
Percentage viability of HaCaT cells with a 24 h treatment with (**a**) 0.01 M phosphoric acid (PA) and (**b**) 0.01 M citric acid (CA), with pH ranges from 3 to 7, followed by a 24 h resting period with DMEM-7 replacement. The viability of HaCaT cells was significantly higher after treatment with 0.01 M phosphoric acid pH 4 and 5 (PA-4 and PA-5). The percentage viability of HaCaT cells was also observed to be statistically significant after treatment of 0.01 M citric acid buffers with pH values of 4, 5 and 6 (CA-4, CA-5 and CA-6). Each bar represents the mean ± standard deviation. * *p* < 0.05, ** *p* < 0.01 and *** *p* < 0.001 indicates that the results obtained were statistically significant from those of the control group (DMEM-7).

Based on the previous findings, a new treatment regimen was employed to study if cell metabolic activity was affected by the different duration of acidification treatment and the resting interval following treatment regimen C as described in Table 1. In summary, the treatment regimen employed a 24 h acidification treatment period before a subsequent 24 h standard culture medium replacement (resting period), and the metabolic activity was measured after every incubation period (24 h). The alteration in the treatment duration for both phosphoric (PA-3 to PA-7) and citric (CA-3 to CA-7) acidic buffers showed a significant improvement of the metabolic activity of HaCaT cells in comparison to that of DMEM-cultured cells (Figure 5). HaCaT cells tolerated the change in pH and showed increases in percentage cell viability of 15.72%, 10.51% and 8.82% at pH 4, 5 and 6 (PA-4 to PA-6) treatments when compared to that of HaCaT cells cultured in DMEM, respectively (Figure 5a). Similarly, increases of 32.79%, 25.21% and 31.72% in percentage cell viability were observed in HaCaT cells when treated with citric acid buffers adjusted to pH 4, 5 and 6 (CA-4 to CA-6), respectively, indicating higher metabolic activity in these treatment groups (Figure 5b). Furthermore, HaCaT cells treated with both phosphoric and citric acid buffers with a 24 h resting period further improved the metabolic activity and percentage cell viability. Phosphoric acid treatment significantly increased cell viability by 44.00%, 45.02 and 25.08% for PA-4, PA-5 and PA-6, and citric acid treatment increased cell viability by 41.28%, 31.98 and 30.80% for CA-4, CA-5 and CA-6, respectively.

**Figure 6:**
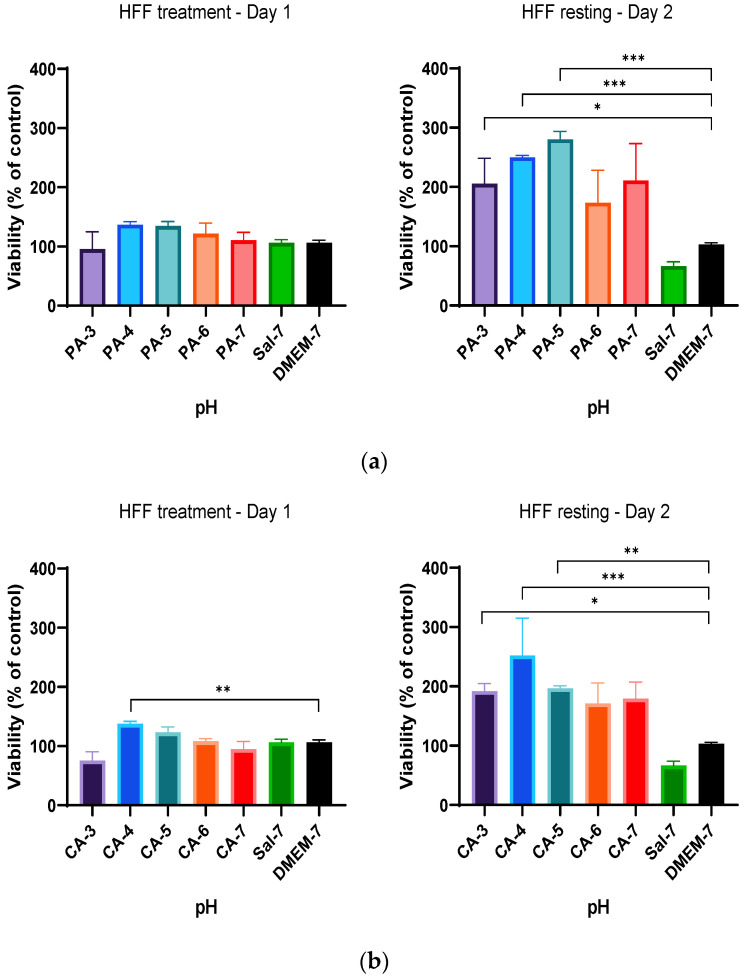
The effect of acidic pH buffers on the metabolism rate of HFF fibroblast cells. HFF cells were cultured in DMEM growth media with the treatment of acidic buffers for a duration of 24 h, followed by aspiration and replacement of fresh DMEM media for another 24 h. (**a**) Percentage viability of HFF cells when treated with 0.01 M phosphoric acid (PA), with pH adjusted to 3, 4, 5, 6 and 7 and (**b**) treatment with 0.01 M citric acid (CA) buffer solutions (pH 3 to pH 7). The results are expressed as mean ± standard deviation. Statistical significance (* *p* < 0.05, ** *p* < 0.01 and *** *p* < 0.001) when compared with the percentage viability of HFF cells in the control group (DMEM-7).

This treatment regimen was repeated on fibroblast (HFF) cells. A similar increment in percentage cell viability of HFF cells was observed when treated with both phosphoric and citric acid buffers. Similarly, initial 24 h acidification treatment with phosphoric acid showed higher-percentage HFF viability of 36.18%, 34.5% and 21.56% at pH 4–6, as shown in Figure 6a. Citric acid also improved the metabolic activity of HFF cells at pH 4, 5 and 6, showing increases of 37.66%, 23.17% and 8.19%, respectively (Figure 6b). After implementing a resting period, significant increases in percentage viability of 73.51%, 149.84% and 180.07% were observed in HFF cells when treated with phosphoric acid buffers with pH adjusted to 3, 4 and 5, respectively, in comparison to that of DMEM-cultured HFF cells (Figure 6a). After a replacement of the culture medium (resting period) and resting interval of 24 h, similarly significant increases of 96.9%, 152.3% and 71.06% were also observed in the metabolic rate of HFF cells when treated with citric acid buffers with pH adjusted to 3, 4 and 5, respectively, as depicted in Figure 6b. These findings demonstrated a time-dependence correlation between metabolic activity of HaCaT and HFF cells and duration of resting interval following the initial treatment. This increased metabolic activity at Day 2 in comparison to that of Day 1 treatment and control group indicated an increase in cell proliferation rate by acidification, influenced by a time-dependent regimen.

### 2.5. Acidification Increases Migration Rate of Keratinocytes and Fibroblasts

**Figure 7:**
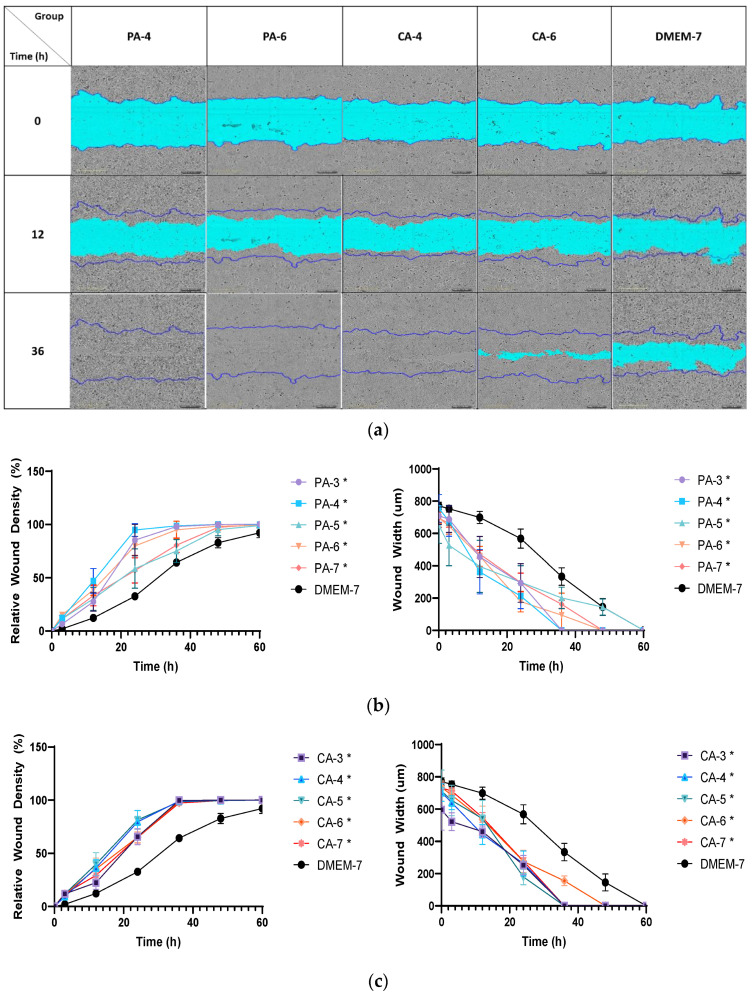
(**a**) Representative contrast images illustrating the migration rate of keratinocyte cells (HaCaT) following acidification treatment regimen C. (**b**) Relative wound density and wound width measured after treatment with 0.01 M phosphoric acid (PA) buffers with pH values of 3, 4, 5, 6 and 7. (**c**) Relative wound density and wound width obtained after incubation with 0.01 M citric acid (CA) buffers. Each error bar represents the mean ± standard deviation. * *p* < 0.05 indicates that the results obtained were statistically significant from those of the control group (DMEM-7).

The effect of both acidic buffers with pH adjusted to 3–7 following treatment regimen C (Table 1) on the rate of migration of keratinocytes (HaCaT) and fibroblasts (HFF) was investigated using a scratch migration assay as described in Section 4.5 (Figure 7a). As shown in Figure 7b, the rate of migration of HaCaT cells was enhanced following treatment with low-ionic-strength (0.01 M) phosphoric and citric acid buffers in comparison to DMEM-cultured cells (DMEM-7). The phosphoric acidic buffer at pH 4 (PA-4) was found to induce the highest cell density (confluency) in the artificial wound gap, which showed 50% cell confluency at T = 12 h followed by 80% confluency at T= 20 h. Other pH showed 50% cell confluency at T = 13–22 h with 80% confluency at T = 22–39 h in comparison to DMEM-cultured cells that reached 80% by T = 50 h. Citric-acid-buffer treatments from pH 3 to 7 induced similar migration of HaCaT cells (Figure 7c), especially pH 5 buffer (CA-5), which achieved 50% HaCaT cell confluency by T = 16 h, with other pH achieving 50% confluency at T = 17–20 h. All pH for citric acidic buffers showed similar 80% confluency at T = 24–28 h.

**Figure 8:**
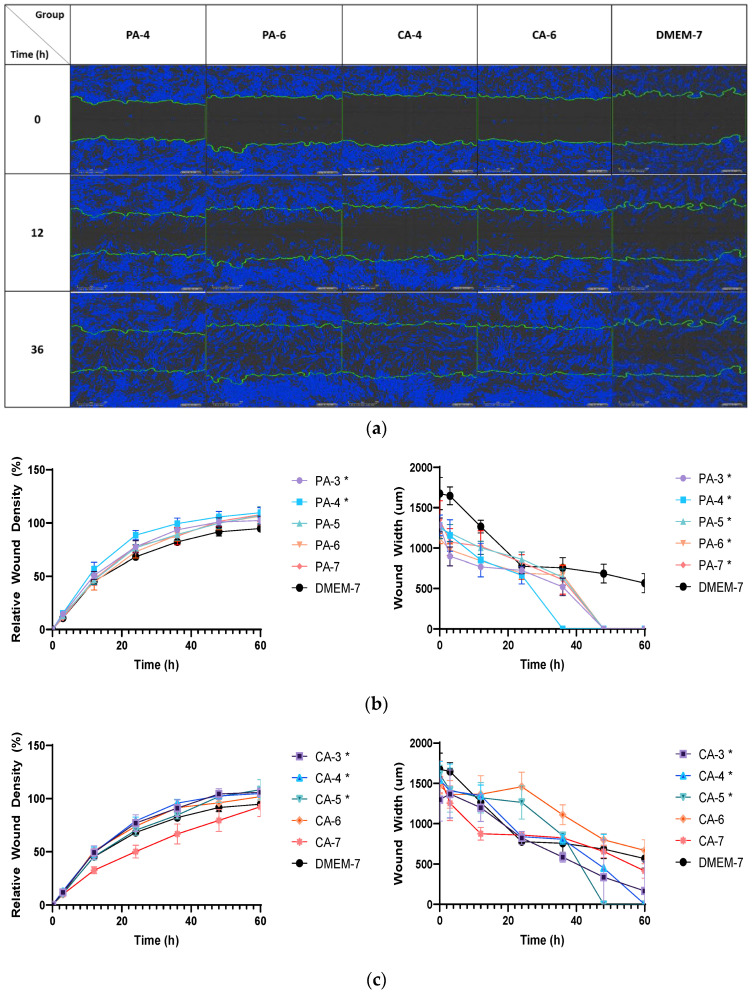
(**a**) The duration taken for HFF fibroblasts to migrate across an artificial open wound when treated with acidic buffer solutions prepared from phosphoric acid and citric acid. (**b**) The percentage relative wound density and wound width measured after a treatment with 0.01 M phosphoric acid (PA) buffers with pH ranges from 3 to 7. (**c**) The percentage relative wound density and wound width of an HFF cell monolayer after incubation with 0.01 M citric acid (CA) buffers with pH adjusted to 3, 4, 5, 6 and 7. Each error bar represents the mean ± standard deviation. * *p* < 0.05 indicates that the results obtained were statistically significant from those of the control group (DMEM-7).

The effect of acidification using phosphoric and citric acid buffers on migration rate was also investigated with HFF fibroblast cells (Figure 8a). A low-ionic-strength phosphoric acid (0.01 M) buffer at pH 4 (PA-4) induced the fastest rate of HFF cell migration, reaching 50% confluency at the wound gap by T = 10 h, and other pH showing it at T = 11–13 h (Figure 8b). In contrast, all pH phosphoric acidic buffers induced a similar migration rate, achieving 80% confluency at T = 21–30 h, whilst the DMEM control group took between T = 42–48 h to achieve 80% confluency for HFF cells. With citric acid, pH 4 (CA-4) was found to show the fastest 50% confluency by T = 13 h, while other pH did it at T = 13–26 h, as shown in Figure 8c. HFF cell confluency of 80% by all pH treatments was obtained beat T = 32–52 h in comparison to that of DMEM-cultured HFF cells that reached 100% confluency after T > 60 h (data not shown).

## 3. Discussion

In this study, phosphoric and citric acids were chosen as model acidic buffers to investigate the effect of acidification of the cell microenvironment on the metabolic activity and migration rate of human immortalized keratinocytes (HaCaT) and human foreskin fibroblasts (HFF). Phosphoric acid was chosen for its important phosphorous constituent that plays a vital role in cell division, growth and development [19,20]. Citric acid is a natural organic acid that acts as an intermediary in the regulation of the tricarboxylic acid cycle (TCA) and plays an important role in the oxidation of carbohydrates, fats and proteins to yield energy for cell proliferation and metabolism [21,22].

A preliminary study of cell viability in HaCaT cells showed that treatment with high-ionic-strength acidic buffers leads to premature apoptosis. Schreml et al. (2014) and Lönnqvist et al. (2015) reported that a short exposure to strong acidic pH reduced cell migration and proliferation by inducing cell apoptosis [23,24]. A similar study by Kruse et al. (2017) found that acidification of a culture medium with high-ionic-strength 0.1 M acetic acid and 1 M potassium hydroxide buffer reduced the proliferation and migration rate of primary keratinocytes and fibroblasts in vitro and reduced the effectiveness of wound re-epithelization and closure in vivo [25]. The findings of cell apoptosis with a high-ionic-strength buffer are consistent with those of other studies reporting a disruption of ions in the culture medium by aggregation-forming crystals [26]. Crystallization can cause structural alterations of triglycerides and free fatty acids in the culture medium and reduction of lipids necessary for the differentiation and proliferation of cells, resulting in cell cycle arrest at the resting phase (G0/G1). Interestingly, our results showed HaCaT cells treated with low-ionic-strength acidic buffers displayed good tolerance and further increased metabolic activity after an initial 24 h acidic treatment (Figure 1), indicating that a low-ionic-strength acid may enhance cell growth.

Different treatment regimens were used to investigate the effect of acidification on cell functions. Regimen A treatment (24 h acidification treatment with no resting period) highlighted the importance of acidifying the cell microenvironment to improve cell metabolic activity. Acidification with phosphoric and citric acid buffers significantly improved the metabolic activity of HaCaT and HFF cells after the initial 24 h acidification treatment at all pH ranges 3–7. However, implementing regimen B treatment, which was a 12 h acidification treatment and a subsequent 24 h resting period, to HaCaT cells showed significantly lower cell viability at low acidic pH 3–5, while both cell lines were unaffected by higher acidic pH of 6 and 7, which were closer to physiological pH (Figure 3). This finding indicates that pH plays an important role in regulating cellular metabolism and growth. Such behavior was reported by a study using manuka honey dressing at acidic pH on chronic wounds. This study showed that reducing wound pH by 0.1 units stimulated wound healing by 8.1%, with dressings at higher pH of 7.8 demonstrating minimal epithelialization and patients with wound pH above 8.0 experiencing increased wound size [27]. Another clinical study by Kaufman et al. (1985) also reported that topical acidification of wounds to pH 3.5 significantly enhanced epithelialization and the closure of burn wounds, whilst alkalinization retarded wound closure and increased the thickness of scar tissues [1]. Other studies have also shown a significant improvement in wound healing properties, including myofibroblasts contraction, fibroblasts migration and DNA synthesis, when the pH of the wound was lowered to an acidic condition [2,28].

Our results showed that changes in treatment duration and resting period affected the metabolic activity of both HaCaT and HFF cells. Employing treatment regimen C, which was a 24 h acidification treatment and a subsequent 24 h resting period, showed not only a significant increase in cell metabolism after the first 24 h treatment (Day 1) but a further increase in metabolic activity than that in the control group after the resting interval (Day 2). This indicated that cell proliferation also increased following this treatment regimen. The importance of treatment duration and a resting interval was further demonstrated with regimen C enabling a significantly faster cell migration rate in both keratinocytes (HaCaT) and fibroblasts (HFF). The ionic strength or molar concentration of hydrogen ions (H^+^) of acid is hypothesized to improve cell proliferation and migration by affecting cell polarity and epithelial potential. Clinical studies showed that acidification of the wound by increasing H^+^ concentration showed improved wound regeneration. This difference in polarity due to H^+^ and epithelial potentials (EP) between wounded and unwounded tissues promoted cellular migration during the tissue proliferation stage [2,29,30].

## 4. Materials and Methods

### 4.1. Chemicals

Dimethyl sulfoxide (DMSO), phosphoric acid, citric acid and sodium hydroxide were purchased from Sigma-Aldrich (Castle Hill, NSW, AUS). Dulbecco Modified Eagle Medium (DMEM), fetal bovine serum (FBS), penicillin–streptomycin, trypsin-EDTA and phosphate-buffered saline (PBS) were purchased from Gibco, ThermoFisher Scientific (Scoresby, VIC, AUS).

### 4.2. Buffer Preparation

Phosphoric acid solutions (H_3_PO_4_, 0.01 M and 0.1 M) were prepared by gently pipetting 0.068 mL and 0.68 mL of 85% w/w phosphoric acid to 25 mL of sterile Milli-Q water, respectively. A mixture of solutions was mixed thoroughly using a magnetic stirrer, and the final volume was adjusted to 100 mL with sterile Milli-Q water.

Citric acid solutions (C_6_H_8_O_7_, 0.01 M and 0.1 M) were prepared by adding 100 mL of sterilized Milli-Q water to crystalline citric acid powders weighing 0.19 g and 1.9 g, respectively. Sodium hydroxide solutions (NaOH, 0.01 M and 0.1 M) were prepared by adding 100 mL of sterilized Milli-Q water to 0.4 g and 4.0 g crystalline sodium hydroxide powders, respectively. All mixtures were mixed thoroughly by sonication before experimentation.

Both phosphoric acid and citric acid buffers were prepared by adjusting pH values of phosphoric acid (0.01 M and 0.1 M) and citric acid (0.01 M and 0.1 M) with 0.01 M and 0.1 M sodium hydroxide solutions to pH values of 3, 4, 5, 6 and 7, respectively. All acidic buffer solutions were incubated for 24 h at room temperature, and the pH values of all the prepared buffers were confirmed with an Orion Star A321 portable pH meter (ThermoFisher Scientific, Scoresby, VIC, Australia) prior to experimentation.

### 4.3. Cell Culture

Human immortalized keratinocytes (HaCaT) and human foreskin fibroblasts (HFF) were purchased from ThermoFisher Scientific (Scoresby, VIC, Australia) and ATCC (Noble Park North, VIC, Australia), respectively. HaCaT and HFF cells were cultured in Dulbecco Modified Eagle Medium (DMEM) supplemented with 10% fetal bovine serum (FBS) and 1% penicillin–streptomycin and incubated at 37 °C with 5% CO_2_. The cells were seeded in T75 flasks at a concentration of 1 × 10^4^ cells/mL. Passaged cells were split 1 in 5 every 3 to 4 days. The cells were collected by trypsinization with a 1× trypsin-EDTA solution (company and country) following final culturing of 7 days.

### 4.4. Cell Viability Assay

**Figure 9:**
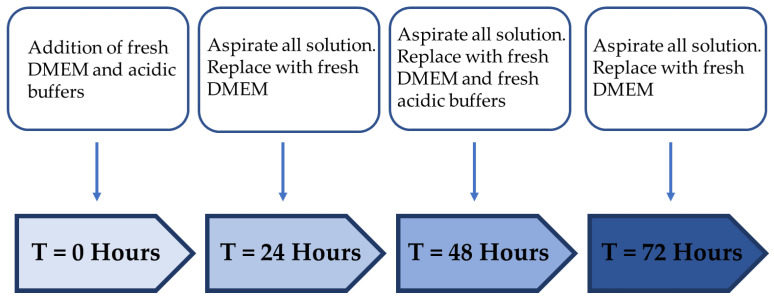
An example of the experimental protocol for treatment regimen C, indicating 24 h of treatment followed by a 24 h resting period.

The cell viability assay that indicated the metabolic activity, cytotoxicity and proliferation of viable cells was determined using an MTT assay. The cells were plated at a density of 1 x 10^4^ cells/well in a 96-well plate and incubated overnight at 37 °C in 5% CO_2_. Following method as described in Figure 9, the growth medium was replaced with fresh DMEM at T = 24 h before the addition of different acidic buffers with various pH values (phosphoric acid pH 3–7 and citric acid pH 3–7). HaCaT and HFF cells were seeded in triplicates and incubated for 24 h. At T = 48 h, the growth media with various pH buffers were aspirated from each well and replaced with fresh DMEM (resting period). At T = 72 h, DMEM was aspirated and replaced with new DMEM with acidic buffers (treatment period). Replacement of DMEM and acidic buffers was carried out for 3 days, during which cell viability was assessed at an interval of 24 h. At a concentration of 2 mg/mL, 50 µL of MTT/PBS solution was added to each well and incubated for 4 h at 37 °C in 5% CO_2_. The wells were replaced with 150 µL of dimethyl sulfoxide (DMSO) to dissolve MTT crystals formed for 10 min at room temperature. The percentage viability of cells was measured by the absorbance of the MTT crystals formed during the experiment at a wavelength of 540 nm in an EnSpire Plate Reader (PerkinElmer, Waltham, MA, USA).

### 4.5. Cell Migration Assay

The cell migration assay assessing the rate of wound coverage in a monolayer of 90% confluent cells was performed in IncuCyte^®^ ZOOM (Essen BioScience, QLD, Australia). The cells were plated at 1 × 10^4^ cells/well in a 96-well flat-bottom ImageLock plate and incubated overnight at 37 °C in 5% CO_2_. Following 24 h of incubation, an artificial scratch wound measuring 700 to 800 µm was applied to the monolayer of 90% confluent cells in each well using a WoundMaker (Essen BioScience, QLD, Australia). The media were aspirated from the wells to remove debris and washed with sterile PBS. A volume of 100 µL of DMEM with 20 µL of acidic buffer solutions was added as treatment and incubated at 37 °C in 5% CO_2_. Live cells were imaged using a phase-contrast microscope with a ×10 objective (Zeiss Axiovert 200; Carl Zeiss, Jena, Germany). Microscopic images were captured at an interval of 3 h for 72 h following the initial scratch (Prior, Cambridge, UK).

### 4.6. Data Analysis

Continuous variables are displayed as the mean ± standard deviation, and categorical variables—as percentages. Statistical analysis between treatment and control groups was carried out using two-way analysis of variance (ANOVA) for multiple comparisons and Dunnett’s multiple comparisons test for independent groups’ comparison. The level of statistical significance was set at less than 5%.

## 5. Conclusions

Our study showed that cell acidification with phosphoric and citric acid buffers enhanced cell proliferation and improved directional cell migration in HaCaT and HFF cells. There was little effect of acidic buffer treatment at low ionic strength (0.01 M) and pH ranges of 4–5, however, a high-ionic-strength acid resulted in reduced metabolic activity and migration, particularly after a subsequent acidification treatment. A time-dependent treatment effect was demonstrated by the inclusion of a resting period after a 24 h treatment with further improvements in cell proliferation and migration observed. This highlights the importance of treatment frequency and duration. Changes in pH and ionic strength suggest that cell proliferation and migration are strongly correlated to the concentration of hydrogen ions (H^+^), which governs the intrinsic activity of cellular activities.

## Figures and Tables

**Table 1:** Summary of treatment regimens.

Treatment Regimen	Protocol	
	Treatment Period (Hour)	Resting Period (Hour)
A	24	-
B	12	24
C	24	24

## Data Availability

Data is contained within the article.
